# A Review of Corneal Transplantation: An Insight on the Overall Global Post-COVID-19 Impact

**DOI:** 10.7759/cureus.29160

**Published:** 2022-09-14

**Authors:** Jay Thakkar, Sandhya Jeria, Aditi Thakkar

**Affiliations:** 1 Ophthalmology, Jawaharlal Nehru Medical College, Datta Meghe Institute of Medical Sciences, Wardha, IND; 2 Public Health, National Institute of Mental Health and Neurosciences, Bengaluru, IND

**Keywords:** cornea storage, corneal blindness, corneal transplantation, covid 19, corona virus disease, keratoplasty

## Abstract

The coronavirus disease 2019 (COVID-19) pandemic made us reframe a lot of the strategies followed in medical practice and ophthalmological services and procedures were also not spared, including corneal transplantation or keratoplasty, the most routine procedure performed worldwide. The prevalence of viral presence in the ocular tissue necessitates a focus on the handling of donor ocular tissue and the functioning of eye banks, ensuring it doesn't risk the patient and the doctor's safety. Restrictions in the movement of people during the pandemic limited the number of donations, causing a shortage of tissues, with a large number of people already waitlisted for tissue needs. The lesson from the COVID-19 pandemic directs us to look for long-term corneal storage techniques taking into consideration the tissue viability time and the possibility of post-pandemic shortages. Although there is not a significant number of reports, the cases of corneal graft rejection post-vaccination against COVID-19 are highlighted and thus should form a part of the lookout while evaluating the possible cause of rejection of grafts. This article summarises the overall impact of the pandemic on corneal transplantation and the possible future of storage techniques, which need to evolve and be adapted.

## Introduction and background

The Wuhan province of China reported outbreaks of pneumonia of unknown aetiology in 2019, which was later named coronavirus disease 2019 (COVID-19) [1]. COVID-19 emerged as a global emergency due to its rapid transmission [2]. The WHO declared COVID-19 a pandemic on March 11, 2020 [3]. Coronaviruses are a group of viruses belonging to the *Coronoviridae* family [4]. The transmission is chiefly due to direct contact, aerosols, or through contaminated objects [5]. Severe acute respiratory syndrome coronavirus 2 (SARS-CoV-2) is a single-stranded positive-sense RNA virus and uses angiotensin-converting enzyme-2 (ACE-2) receptors for cellular entry with the help of its glycoprotein spikes. Research has shown that the virus is continuously mutating significantly in asymptomatic carriers [4]. The COVID-19 outbreak was a time of exceptional chaos, overburdening the healthcare system, in response to which healthcare policies were reframed, thereby ensuring smooth flow of service to the patient.

Corneal transplantation and keratoplasty

The cornea is a six-layered structure [6]. There are numerous corneal pathologies which may be even nutritional and traumatic. Corneal pathologies are one of the important cause of blindness, second only to cataract [7]. Corneal blindness is the third primary cause of blindness globally, the first and second being cataracts and glaucoma, respectively. In these cases, keratoplasty forms the only basis for restoring vision, especially in the case of total corneal decompensation. As a result, keratoplasty remains one of the most routinely performed surgical procedures after cataract surgeries [3]. Keratoplasty has great success rates due to lymphatic and vascular deprivation [8]. Corneal transplant is a novel success procedure implicated in a reversal of corneal blindness [9]. The chief factor in ensuring corneal viability is ensuring viable endothelium since these cells have limited proliferation in situ as they are arrested in the G1 phase of the cell cycle [10].

**Figure 1:**
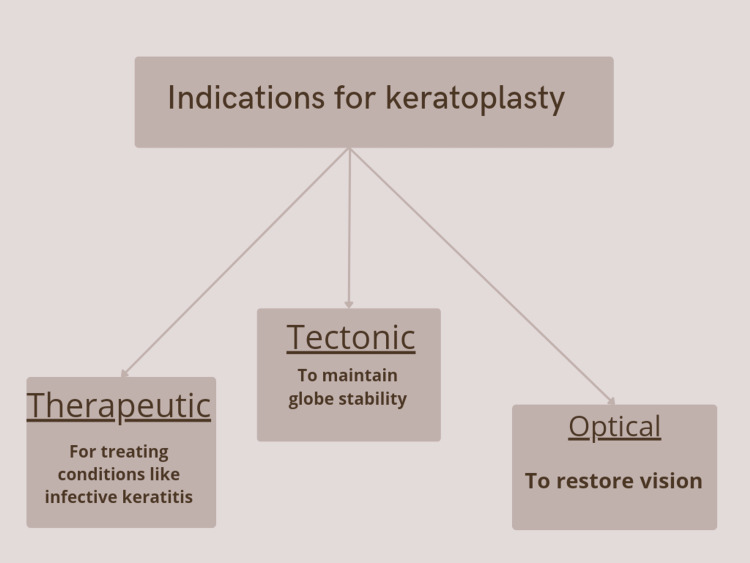
Classification of Keratoplasty Based on Indications

The first corneal transplant procedure was attempted using a rabbit donor cornea by Von Hippel but the first successful one was performed in 1905 by Zirm [6]. Based on indications, keratoplasty is classified into: therapeutic as in infective keratitis, tectonic to maintain the globe stability, and optical to re-establish vision (Figure 1). Also, it can be classified based on different techniques used like penetrating and lamellar keratoplasty, which is of two types: anterior or posterior [6]. The method of selective replacement of a particular part in keratoplasty (lamellar keratoplasty) has significant advantages in terms of complications and graft rejection. Anterior lamellar keratoplasty (ALKP) can further be of three types: superficial, if it involves 30-40% of corneal thickness, automated lamellar therapeutic keratoplasty (ALTK), if the opacity is in the mid-stroma, and deep anterior lamellar keratoplasty (DALK), if the opacity is deeper. Posterior lamellar keratoplasty is done in case of endothelial involvement. There is a broad range of indications of keratoplasty, the chief being bullous keratopathy in developed and infective keratitis in developing countries [6].

The indications of corneal transplantation vary geographically and according to the socio-economic status and demographic characteristics of the country [11]. Corneal transplant surgeries have shifted from complete thickness transplants to replacing only specific corneal layers in the recent past [10]. Corneal transplant has an overall success rate of 65-95% [12]. The Government of India has laid down the target to reduce the blindness prevalence by 0.25 per 1000 cases by the year 2025 [12]. India had shown a significant drastic drift in improved collection and utilization of corneal transplants post the establishment of a Regional Institute of Ophthalmology, Chennai, in 1947. Since then, India has remained at the second position in terms of procurement and transplantation of cornea. The chief contributing states in this included Andhra Pradesh, Telangana, Gujarat, Karnataka, Maharashtra, and Tamil Nadu with an overall utilization rate of 50% in the country with primary reliance and preference for short-term storage techniques of up to 14 days and microbial keratitis being the most common indication for the same in the country [12]. Of the different options in corneal transplantation, the chances of rejection are least with the Descemet membrane and endothelium, following Descemet's membrane endothelial keratoplasty (DMEK). However, there are reports of DMEK corneal graft rejection occurring post-COVID-19 vaccinations, pointing out some probable associations with it [13].

## Review

The scenario

Many healthcare services were curbed during the COVID-19 pandemic including various ophthalmological procedures of which keratoplasty or corneal transplantation was a part. The fact that it is a procedure that can be delayed to a significant time remains one reason and the health force being diverted to serve as the workforce for COVID-19 patient care is another reason why keratoplasty was brought to a halt [14]. In addition, donor screening procedures, which need to be fulfilled before acquiring donor tissue or organ, are cited as another reason for the curb [3]. The suspension of corneal donations and the widening of the corneal demand-supply gap has necessitated the adaption of long-term storage strategies to avoid wastage and prepare for possible uncertainties [12].

Ocular transmission of COVID-19

The SARS-CoV-2 virus enters tissues through ACE-2 receptors after cellular protease priming through transmembrane serine protease-2 (TMPRSS-2). Studies show that both ACE-2 and TMPRSS-2 have a significant expression on both cornea and conjunctiva epithelium; hence, graft acquisition for the transplant remains a point of concern [8]. Though not an emergency surgically, a corneal transplant, based on donor availability becomes a quick-to-act procedure [9]. Barriers in corneal donation during the COVID-19 pandemic caused significant shortages for future surgeries [14]. Most study results are indicative of low transmission through ocular secretions [15]. The interaction between the viral particles with the cellular components by autophagy has a role in viral entry and cellular replication [16]. Despite there being no evidence of the fact that keratoplasty can cause transmission of COVID-19 and the absence of viral RNA in the corneal samples of the patients who died of the disease, corneal donations of COVID-19 patients were deferred. However, the global issue of the low donor-to-demand ratio for tissue needs to be looked as well [3]. There are reports of COVID-19 causing conjunctivitis and viral presence in tear secretions. The tendency of the virus to infect the ocular tissue has an impact on how it is going to affect eye banking globally [8]. There are conflicting reports about the presence and replication of the SARS-CoV-2 virus in the eye tissue with viral particles being detected in COVID-19-infected patients. However, others report no such viral presence in the post-mortem eye tissues of infected patients [17]. Miner et al. reported that there is no significant risk of viral replication in the explant of the cornea and, hence, there are no significant risks associated with corneal transplantation [17].

Sawant et al. studied the presence of SARS-CoV-2 virus in the corneal tissue in 20 eyes, of which 10 were positive for COVID-19 RNA and three had ocular involvement with a positivity rate of 5% for the anterior and 25% for the posterior corneal surface [15]. Though it looks like a negligible risk, the total risk cannot be ruled out. In a study by D’Souza et al., there was a significant prevalence of asymptomatic COVID-19 infection among corneal donors [18]. Whether the virus reaches conjunctival tissue in a retrograde fashion along the lacrimal duct or it is due to primary ocular infection is unclear [19]. Overall studies report the involvement of conjunctiva in COVID-19 patients with the reports of conjunctiva inflammation in many but few positive RT-PCR swab reports, probably due to low viral load or the presence of non-infective RNA fragments. In addition, the tear film has lactoferrin, an antimicrobial protein that inhibits the interaction of the virus with the ACE-2 receptors.

Eye bank guidelines during the pandemic

The function of the eye bank is to harvest, evaluate, preserve, and distribute corneal tissue [20]. The Eye Bank Association of America (EBAA) and other central eye banks restrict donations from COVID-19-infected individuals or from those who had close contact with infected individuals. Double disinfection with povidone-iodine before retrieval and polyvinylpyrrolidone solution can inactivate viruses like SARS-CoV-2 before storage. The guidelines vary with geographies like a need for negative PCR test of the nose, throat, and endotracheal swabs along with serological tests in the United Kingdom and a CT lung for qualified donors in China [8]. Due to the revision of rules for obtaining corneal grafts by EBAA, there has been a drop of about 24-29% in the donors. As per a global survey data between August 2012 and August 2013, and findings published in 2016, there is only one cornea per 70 corneas needed [21]. The transition to post COVID-19 era of transplantation demands interdisciplinary coordination among eye banks and surgeons, and patient education about preoperative delays and on-time cancellations. Thus, enforcement of the new reframed policies for corneal donation between various disciples, the eye banks, surgeons and the patients, needs to be made so as to ensure that the corneal donation procedures continue smoothly. Subsequently, the overall cost of corneal donation may increase because of obvious reasons [8]. In India, as of 2020-21, there was a significant drop in corneal collections to 18,359, which is a 73% drop in comparison to that in 2016-19, building an extensive wait list of people who are in need [22]. Globally, the percentage of corneal transplants dropped by 81% in April 2020 in comparison to the same period in 2019 [9].

Vaccine and graft rejection

The COVID-19 vaccine ChAdoX1 n CoV-19 induces a strong immune response, and there are reports of acute allograft rejection post-vaccination [23]. The presence of class II major histocompatibility complex (MHC) antigens in all the layers of the cornea may trigger its rejection in response to COVID-19 vaccination. The rampant rise in COVID-19 vaccination has induced a debate about total thickness keratoplasty [24]. In patients with keratoplasty, increasing the topical steroid use pre- and post-COVID-19 vaccination may help reduce the possibilities of graft rejection [25]. Despite binocular transplant, a report of uniocular rejection is reported after COVID-19 vaccination [26]. This may be attributed to the fact that it was a repeat graft with a higher chance of rejection. It is better to delay the corneal transplant by three to six months post the second dose [26]. There is a report of diminished vision post keratoplasty after two days of vaccination with ChAdOx1 nCoV-19 recombinant vaccine, Covishield, following the first dose that responded well to steroid-treated immunosuppression restoring graft clarity. This is attributed to the strong immune response induced by vaccination and, therefore, there stands a risk of graft rejection. There is not a significant number of reports, but corneal surgeons need to keep this kind of event as a possibility in mind [23]. There is a need for continuous follow-up of patients who have undergone any ophthalmological procedure and have developed ocular events like eyelid erythema and rash over eyelids, which are easily seen. In patients with corneal transplants, graft rejection presenting as diminished vision, photophobia, redness, and graft edema can be seen. There is debate on second dose vaccination timing post transplantation [27].

The impact

During the pandemic, the Eye Bank Association of India (EBAI) restricted the retrieval, storage, and transport of cornea tissues [28]. However, to fulfil the corneal needs, the National Program for Control of Blindness and Visual Impairment (NPCB-VI) took the initiative to start the hospital cornea retrieval programme (HCRP) [28]. There was a drastic decline in elective keratoplasty but the emergency procedures continued [29]. The requirements in corneal transplantation differ in multiple aspects in comparison to other transplant procedures, especially in regard to the recipient [9]. In India, post COVID-19 lockdown in March 2020, there was a drop in corneal retrieval and drift from short and intermediate-term preservation techniques using McCarey-Kaufman (MK) medium and cortisol, respectively, to long terms ones using glycerol at minus 80 degrees Celcius with a halt on voluntary donations [30]. Parekh et al. reported that between 2020-21 during the time of lockdown, there was a remarkable fall in the corneal grafts [22]. The total demand for corneal transplantation for India is 35% of the total global donations; however, the procure rate remained at only 0.34% during 2018-19, which during the COVID-19 pandemic showed a 73% drop in 2020-21 when compared to 2016-2019 data [22]. There is a heavy rise in demand for corneal tissue post lockdown, according to reports. Looking into the scenario, there appears to be a need for expanding the eye bank network with newer government policies.

In response to the pandemic, the Government of India's Ministry of Health and Family Welfare (MoHFW) and the Indian Council of Medical Research (ICMR) had to devise newer guidelines; these included quarantining the collected tissues for 48 hours before supply for usage, resuming eye banking through hospital corneal retrieval program, no eye banking in containment zones, use of personal protective equipment and reverse transcriptase-polymerase chain reaction (RT-PCR) test of the deceased donor as precautionary measures [31]. The eye bank association restricted corneal tissue retrieval from a person who was or is positive for COVID-19, had acute respiratory fever or illness, acute conjunctivitis, has acute respiratory distress syndrome (ARDS), pneumonia, or whose CT lung shows ground glass opacities. Phylactou et al. showed that despite the cornea being immune privileged, there is an association between the corneal graft rejection post-vaccination with BNT162b2, which causes activation of both humoral and cellular responses where Interferon-gamma producing CD-4 helper T-cells have a crucial role in allograft rejection [13]. In the United States, there was a drop in both the number of donors and referrals by 45% and 31%, respectively, based on a four-month comparison in 2020 and 2019, which was mainly due to restrictions at different places and the revised EBAA guidelines [17]. Voluntary corneal donations are the main source of cornea in India. The year 2020 stands as a dull year in terms of the efforts to eradicate corneal blindness in India [28]. Reports point out that patients who underwent emergency keratoplasty presented late to the hospital post rejection of the graft due to hampered transport, fear of acquiring COVID-19, and unavailability of ophthalmologists. This impacts the further viability of the graft because the earlier the rejection is treated, the better the prognosis. More the delay, the higher the chances of corneal decompensation and graft failure [32]. Though viral RNA. can be detected in the conjunctival tear film, it is suspected that the virus confines itself just onto the surface, increasing the chances that it gets sterilized with povidone-iodine applied to the surface after excision. Still, povidone-iodine doesn’t achieve complete sterilization and thus doesn’t rule out the presence of the virus [33].

What next?

Various long-term corneal preservation techniques may have a significant impact on the future of corneal preservation (Table 1).

**Table 1:** Complications Associated With Various Corneal Preservation Techniques

Preservation technique	Principle	Complications
Cryopreservation	Crystallization of extracellular water	Requires expensive instrumentation techniques, danger of damage to endothelium and affects the transparency
Vitrification	Glass transition	Procedure is technically demanding.
Glycerol preserved	Dehydration of corneal tissue	Prolonged time is required for epithelization of graft and oedema may occur post transplantation
Lyophilised/freeze dried	Compaction of collagen fibres	Opacities may develop after transplantation
Gamma irradiated	Irradiation by cobalt-induced gamma radiation	May affect DNA content of the corneal tissue

Cryopreservation

This involves freezing the corneal tissue at a low temperature of -196 degrees, which causes the crystallisation of extracellular water and also increases its electrolytes leading to osmotic shrinkage of cells. Cryoprotectants are added to prevent thermal trauma to the cells. However, endothelial cells may get damaged, affecting the corneal transparency [12].

Vitrification

This involves the direct conversion of liquid to solid without crystallisation, called glass transition. This is achieved by the addition of some viscous substances and ensures continued random movements of molecules like a liquid although it assumes properties of a solid [12].

Glycerol Preserved Corneas (GPC)

Glycerol is a hygroscopic, colourless, viscous liquid that dehydrates the corneal tissue and has antimicrobial properties. It permits corneal storage for a prolonged duration of 5-23 years. These favourable variables provide us with possible exploitable benefits, especially in emergencies like COVID-19 when there is no regular supply of donors or in conditions of limited eye bank functioning, mainly in countries where corneal tissue is not readily available [12]. Different authors have reported varying results in terms of outcomes with glycerol-preserved corneas [34].

Lyophilised/Freeze-Dried Cornea

It is a dehydration process that involves lowering the vapour pressure after freezing the tissue followed by sublimation of the ice. This process causes the compaction of collagen fibres. This procedure is chiefly used in ALKP for corneal scars and keratoconus [12].

Gamma-Irradiated Sterile Cornea

This includes sterilising corneal tissue grafts against contamination by microbes like viruses, bacteria, and fungus using Cobalt-60-induced gamma radiation, providing a room storage time of up to two years. However, the collagen density and amount of DNA in the gamma-irradiated cornea are low compared to fresh corneal tissues [12].

However, despite available techniques, the use of long-term storage techniques is limited because of an overall depleted level of viable cells in the tissue leaving behind an acellular corneal stromal focus. In addition, these grafts cannot be used for optical purposes, the time for epithelisation of the grafts is long, and there occurs edema and opacification of grafts after transplantation. Contrary to this, there remain some exploitable advantages. These include the lack of MHC antigens, thereby reducing the chances of immune rejection. The acellular cornea can get re-epithelized and regain the corneal structure and function, and most importantly, it acts as an emergency reserve of the cornea, especially for lamellar procedures [12].

In the COVID-19 era, glycerol storage methods are a good option because of their broad possible storage conditions with improved storage at lower temperatures. Also, glycerol is cheaper and readily available, unlike lyophilised corneas, which are expensive to process and difficult to handle [12]. A study by Gupta et al. compared the therapeutic success of the ability to maintain eyeball integrity of fresh corneal transplants (FCT) with GPC on patients who had corneal perforations or infections requiring immediate transplantation [35]. The report showed that both have almost equal efficacy in maintaining ocular integrity with manageable complications and thus GPCs stand as one of the most acceptable options for FCTs when not available.

## Conclusions

Corneal blindness is one of the most demanding procedures regarding corneal grafts requirement. Corneal transplantation forms one of the most effective treatment modalities for the treatment of various corneal pathology. The COVID-19 pandemic had a devastating impact on the functioning of corneal retrieval and donation procedures, the performance of eye banks, as well as performance of ophthalmological procedures. The hit on the eye banks may pose a future problem of corneal shortage due to a sudden fall in how the eye banks originally retrieved the donor corneas. The drop in the number of retrieved corneal tissues needs adaptation of long-term storage techniques while also keeping in mind future requirements. The impact of COVID-19 vaccination on the failure of graft acceptance needs to be thoroughly investigated and more studies need to be carried out to establish a firm conclusion on how COVID-19 vaccination affects corneal grafts. There is a need to redesign policies on the functioning of eye banks with regard to graft-acquiring strategies post the COVID-19 pandemic, thereby ensuring a smooth supply of corneal tissues to meet the demand. Further, development of more long-term storage techniques for corneal tissues remains the need of the hour. 
